# A Cross-Sectional Study of Sex-Specific Associations of Renin and Electrolytes on the Development of Hypertension

**DOI:** 10.3390/jcm15020643

**Published:** 2026-01-13

**Authors:** Seong Beom Cho

**Affiliations:** Department of Biomedical Informatics, College of Medicine, Gachon University, 38-13, Dokgeom-ro 3 Street, Namdong-gu, Incheon 21565, Republic of Korea; sbcho1749@gachon.ac.kr

**Keywords:** blood pressure, hypertension, sex, renin, sodium

## Abstract

**Background/Objectives**: Blood renin and electrolyte levels are associated with blood pressure and hypertension. While sex-specific effects of such factors have been investigated, exact comparisons of the factors between the sexes have been scarce. **Methods**: Using cohort data from the Korean Genome and Environmental Study (KoGES), the study population that did not receive any interventions for blood pressure was determined. Blood levels of renin and electrolytes, including sodium, potassium, chloride, and calcium, were used to test their relationship with hypertension and blood pressure. Confounding variables, including age, body mass index (BMI), waist-to-hip ratio, family history of hypertension, alcohol consumption, smoking, blood urea nitrogen, creatinine, protein, and albumin levels, were used for adjustment in the multiple regression analysis. **Results**: In the single-variable analysis, sodium levels were significantly higher in the female population, and showed strong associations in the multiple regression analysis. Blood potassium levels showed no significant sex-specific differences. Among these factors, renin showed the greatest significance in both the total population and sex-specific groups. Moreover, in the development of hypertension, the effect size of renin was significantly different between sexes. Additionally, BMI tended to show stronger associations in females. **Conclusions**: This study identified sex-specific differential effects of renin and other electrolytes that are important in the pathophysiology of blood pressure. These findings provide clues for the more precise management of hypertension.

## 1. Introduction

Hypertension is a major global public health issue, affecting approximately 1.28 billion people as of 2023 [1]. As hypertension leads to many cardiovascular complications, it should be controlled or prevented in its early phase [2]. Therefore, identification of risk factors is essential in controlling this disorder.

The renin–angiotensin–aldosterone axis (RAAA) is critical to the development of hypertension [3]. While the RAA system maintains the homeostasis of blood pressure, it can cause hypertension under pathologic conditions such as hepatorenal and cardiorenal syndromes [4,5]. For example, angiotensin II causes an abrupt increase in portal pressure and is involved in hepatic cell proliferation in cirrhosis [4]. Moreover, it stimulates insulin resistance, mitochondrial dysfunction, and proinflammatory cytokine production in NAFLD [4]. In cardiorenal syndrome, the RAAS causes oxidative stress by promoting the generation of reactive oxygen species (ROS) and the oxidation of low-density lipoprotein (LDL), which results in atherosclerosis through the stimulation of cell adhesion molecules such as VCAM-1 and P- and E-selectins [5]. Additionally, the RAAS causes constriction of glomerular arterioles, inducing the production of FGF23, which is involved in uremic toxin retention and renal fibrosis [5]. Sodium and potassium are the principal electrolytes involved in the regulation of blood pressure, and blood electrolyte levels are controlled by the renin–angiotensin–aldosterone axis [6]. In addition to sodium, potassium and chloride also known to be associated with hypertension [3,7]. Therefore, dietary control of these factors is an important strategy for controlling hypertension [8,9]. In addition to electrolytes, other factors such as body mass index (BMI) [10,11], waist-to-hip ratio (WHR) [12,13], and family history are well-known risk factors for hypertension.

Although these factors have been validated in the general population, some studies have suggested sex differences in their effects on hypertension. For example, biological mechanisms of salt-sensitive blood pressure identified in population studies have been investigated [14]. A computer simulation revealed that a higher intake of potassium could reduce blood pressure in women [15]. Other studies have shown that BMI and WHR have differential effects on the development of hypertension according to sex [16,17]. These results indicate that demographic and clinical factors of hypertension have sex-specific effects.

In this study, the sex-specific effects of demographic and biochemical factors known to be associated with hypertension were investigated using cohort data from the Korean population. The effects were evaluated in terms of the hypertension status, systolic blood pressure (SBP), and diastolic blood pressure (DBP).

## 2. Materials and Methods

To identify the sex-specific effects of renin and electrolytes, including sodium, chloride, potassium, and calcium, epidemiologic and biochemical data from the Ansan-Ansung (ASAS) cohort were used. The ASAS cohort is part of the Korean Genome and Epidemiology Study (KoGES) project that was developed in 2002 to address public health issues in terms of personalized and preventive health care [18]. The project comprised three main cohorts: ASAS, Cardiovascular Disease Association Study (CAVAS), and Heath Examinee (HEXA). The ASAS cohort was characterized by recruiting participants from two cities (Ansan and Ansung) located in Kungki-do. Of the three cohorts, the ASAS cohort had the longest follow-up duration of 20 years. At baseline, epidemiologic data including sex, age, BMI, WHR, family history of hypertension (fhHTN), SBP, DBP, history of diagnosis, and treatment for hypertension were obtained. Plasma levels of renin and four electrolytes (sodium, chloride, potassium, and calcium) were obtained from the cohort data. This study was performed using a cross-sectional design; therefore, demographical, and biochemical data obtained at the baseline of follow-ups were used.

To identify the linear relationship between the factors and target variables (hypertension status, SBP, and DBP), T-test and Pearson’s correlation coefficient test were applied. These tests were applied to the data for electrolyte and renin levels and were performed independently for male and female populations. Multiple regression analysis of hypertension status, SBP, and DBP was performed with adjustments for possible confounding variables retrieved from the cohort data. After fitting the regression lines, effect sizes (beta coefficients) of the variables were tested. It is possible that multiple statistical testing causes false-positive results. To control for this possibility, multiple testing correction was performed using the Bonferroni’s method, in which the adjusted *p* value is determined by dividing alpha by the number of statistical tests. The significance level of alpha was set to 0.05, which is the same as the nominal *p* value threshold.

When statistical tests were performed on male and female populations, it is possible that the results of the statistical tests were simultaneously significant in both sexes. Under these conditions, the results were compared using permutation tests. The permutation test was based on the random shuffling of male and female samples, and the *p* value of the test was determined by counting the instances in which the difference in statistics between shuffled populations was greater than the original difference in the statistics. The number of permutations was set to 10,000, considering the adjusted *p* values in multiple testing corrections.

## 3. Results

### 3.1. Comparison of Renin and Electrolytes Between Normal and Hypertensive Patients

From the ASAS cohort data, 10,030 participants were included in the baseline examinations in 2001 and 2002. The number of male and female participants was 4758 and 5272, respectively. Participants with diabetes and hypertension were excluded based on their diagnosis or medication history (*n* = 2301). Of the remaining participants, those with missing values for any of the variables were excluded. Consequently, 7729 participants were included in the analysis. Based on the hypertension criteria of (1) systolic blood pressure ≥ 140 mmHg or (2) diastolic blood pressure ≥ 90 mmHg, 1237 patients were considered hypertensive. All variables used in this analysis were compared between the normal and hypertensive groups using statistical tests. Table S1 shows the results of the comparison between the normotensive and hypertensive groups in the total population. As expected, several variables (age, sex, BMI, WHR, SBP, DBP, albumin, protein, BUN, creatinine, smoking, and alcohol consumption) known to be differentially distributed between healthy and hypertensive patients showed significant results (Table S1).

Electrolytes levels were compared using sex-specific datasets. In the male population (*n* = 3733), sodium levels showed no significant difference between the normal and hypertensive groups, whereas significant differences were observed in the total population (Table 1). However, the female population showed a significant result. Except for potassium and chloride, all variables were significant in the total population (Table 1).

### 3.2. Correlation Between Blood Pressures and the Other Variables

To determine the linear relationship among renin, electrolytes, and blood pressures, Pearson’s correlation coefficients (CCs) were determined. The adjusted *p* value was determined to be 1.67 × 10^−3^ (=0.05/30) according to Bonferroni’s multiple testing correction. In the total population, three variables (sodium, calcium, and renin) were significantly correlated with both SBP and DBP (Table 2). Potassium was significantly correlated only with diastolic pressure, whereas chloride showed no significant correlation with systolic or diastolic pressure (Table 2).

In the male population, the results differed from those of the total data analyses. Although highly significant correlations were observed between sodium and systolic or diastolic blood pressure in the total population, no significant correlations were observed in the male population. The CC between systolic blood pressure and sodium was 0.08 in the total population but was reduced to −0.03 in the male population. In case of diastolic pressure in the male population, the correlation decreased from 0.11 to 0.02. Potassium levels showed no significant correlation with systolic or diastolic blood pressure (Table 2). Although chloride levels showed no significant correlations in the total population, they exhibited a significant correlation with SBP in the male population (−0.06, *p* = 2.84 × 10^−4^). Calcium levels were not significantly correlated with diastolic pressure in the male population (CC = −0.04, *p* = 1.09 × 10^−2^), unlike the total population (CC = 0.06, *p* = 4.19 × 10^−8^).

In the female population, the correlations between blood pressures and variables were consistent with those in the total population. Correlations with potassium and renin levels showed clear differences between the sexes (Table 2). Potassium levels in the female population showed a significant correlation with DBP, whereas the correlation was not significant in the male population (CC = −0.02, *p* = 2.12 × 10^−1^). For renin, the magnitudes of the correlations were different. In general, this correlation was more pronounced in the female population (Table 2). The correlation between SBP and renin in the female population (CC = −0.17, *p* = 3.80 × 10^−26^) was 2.43-fold of the correlation in the male population (CC = −0.07, *p* = 3.02 × 10^−5^), and the difference increased in correlation between DBP and renin; the correlation in the female population (CC = −0.12, *p* = 1.61 × 10^−15^) was 4-fold of that in the male population (CC = −0.03, *p* = 5.00 × 10^−2^).

### 3.3. Multiple Logistic Regression Analysis of Renin and Electrolytes with Adjusting Clinical Factors

In this study, covariates for the multiple regression analysis were selected from the blood biochemical tests. Specifically, factors known to be associated with hypertension were considered. Additionally, variables that showed statistical significance when comparing the normal and hypertension groups were selected for the regression analysis (Table 1 and Table S1). The variables included age, sex, BMI, WHR, albumin, BUN, creatinine, protein, calcium, sodium, potassium, chloride, renin, smoking, alcohol consumption, and fhHTN. Although fhHTN was not statistically significant in the univariate analysis (*p* = 0.17, Table S1), it was included because the genetic effects on hypertension are well-established. To identify the hypertension-inducing effects of renin and electrolytes, considering confounding variables, multiple logistic regression analysis was performed with the 16 selected variables. Table S2 shows the results of the logistic regression analysis for hypertension and the selected variables. After analysis, nine variables, including age, sex, BMI, WHR, albumin, protein, sodium, renin, and fhHTN were found to be significant with Bonferroni’s multiple testing correction (adjusted *p* value = 3.12 × 10^−3^). Of the result, WHR had the greatest effect size (beta = 4.28, *p* = 2.47 × 10^−15^), and albumin had the second greatest effect size (beta = 0.44, *p* = 2.68 × 10^−3^).

In the male population, four variables (age, BMI, WHR and alcohol consumption) showed a significant effect size in the multiple logistic regression analysis (Table 3). While no significance was observed in female population, alcohol consumption showed a significant effect size (beta = 0.49, *p* = 8.81 × 10^−5^). Although sodium had a significant effect size on the total population (Table S2), it showed no significant effect size in male population (Table 3). When multiple logistic regression was applied to the female population, five variables (age, BMI, WHR, sodium and renin) showed significant effects (Table 3).

### 3.4. Estimation of Effects of Renin and the Electrolytes on Systolic and Diastolic Blood Pressure with Adjusting Clinical Factors

In the multiple linear regression analysis of SBP and other variables, eight variables, including age, sex, BMI, WHR, sodium, potassium, renin, and family history of hypertension had significant effect sizes in the total population (Table S3). Of the variables, WHR had the greatest effect size (beta = 36.24, *p* = 1.74 × 10^−38^). While chloride showed no significant result in the correlation analysis, it showed significant effect size in the regression analysis (beta = −0.33, *p* = 2.11 × 10^−4^). Table S3 presents the results of the multiple linear regression analysis of SBP and the selected variables. Although the *p* value of BMI was significant, it had a relatively small effect size of the significant variables.

In the male population, age, BMI, WHR, protein, renin, alcohol consumption, and family history of hypertension showed significant effect sizes in the regression analysis of SBP (Table 4). Interestingly, alcohol consumption (beta = 2.45, *p* = 6.94 × 10^−5^) had a significant effect on SBP, which was not significant in the total population. Multiple linear regression analysis of the female population also showed that age, BMI, WHR, protein, renin, and family history of hypertension levels were significant (Table 4). In addition, sodium levels were significant only in the female population (beta = 0.44, *p* = 8.08 × 10^−4^). Interestingly, alcohol consumption showed a significant result in the male population, but it had no significance in female population (beta = −0.73, *p* = 1.70 × 10^−1^).

Table S4 presents the estimated effect sizes and their corresponding *p* values from the multiple linear regression analysis of DBP and other variables. In total, twelve variables (age, sex, BMI, WHR, albumin, BUN, protein, sodium, chloride, renin, alcohol consumption, and fhHTN) showed significant results. In the male population, nine variables (age, BMI, WHR, albumin, sodium, chloride, renin, alcohol consumption, and fhHTN) showed significant results, whereas six (age, BMI, WHR, albumin, sodium, and fhHTN) showed significant results in the female population. Interestingly, alcohol consumption also had no significant effect on the total population but was significant in male population (Table 5). Alcohol consumption was significant only in the male group (beta = 2.07, *p* = 8.86 × 10^−7^).

### 3.5. Identification of Differential Effects of Hypertension-Associated Demographic and Biochemical Factors Between Sexes

In the comparison of the effects between the sexes, some factors showed discordant significances. However, several factors showed differences in effect sizes between sexes, although showing significances in both sexes. For such cases, permutation tests were performed to determine whether the differences were significant.

In the T-test, calcium showed significant results in both sexes. Therefore, permutation-based test was conducted. First, t-statistics for comparing calcium levels between normal and hypertension groups were evaluated whether the differences in the t statistics between sexes were significant. With 10,000 permutations, the difference in T-statistics between sexes was not significant (*p* = 3.83 × 10^−1^). The t-statistics from the correlation coefficients between SBP and renin levels showed significant differences between the male and female groups. In the permutation test, no single cases exceeded the original differences between the T-statistics of the male and female groups in the correlation analysis of SBP and renin levels (*p* < 0.0001).

When multiple logistic regression analysis was applied, age, BMI, and WHR showed significant results in both sexes. When the permutation test was applied, however, none of the variables showed a statistical significance. The permutation *p* value of age, BMI, and WHR were 0.11, 0.06, and 0.11, respectively.

In multiple linear regression analysis of SBP, age, BMI, WHR, protein, renin, and fhHTN were significant factors in both sexes. Of the variables, only the BMI showed a significant result in the permutation (*p* = 1.00 × 10^−3^). The age (*p* = 1.10 × 10^−2^) and renin (*p* = 1.30 × 10^−2^) showed nominally significant results but did not reach the threshold *p* value of multiple testing correction (adjusted *p* value = 0.05/6 = 8.33 × 10^−3^). In the DBP analysis, six variables (age, BMI, WHR, albumin, sodium, and family history of hypertension) were significant in both sexes (Table 5). The permutation test identified that the effect sizes of age and BMI were significantly different between the sex groups (*p* = 6.40 × 10^−3^ and 4.00 × 10^−3^, respectively). The remaining WHR, albumin, sodium and fhHTN levels showed no significance, even nominally (*p* < 0.05) or after Bonferroni’s multiple testing correction (*p* = 0.008).

## 4. Discussion

In this study, the sex-specific effects of renin and electrolytes including sodium, potassium, chloride, and calcium on the development of hypertension and blood pressure were investigated using population data. Although significant associations were identified in the total population, some factors showed significant differences between sexes. Since all analyses were performed on participants who had no history of diagnosis or treatment for hypertension, the results seemed to be representative of the natural pathophysiology of hypertension.

During the selection of the study population, patients with diabetes were excluded due to the potential use of angiotensin-converting enzyme (ACE) inhibitors, which could introduce bias into the analysis. Because the cohort data lacked specific medication records, all diabetic patients were removed. However, the results remained consistent even when these patients were included. The statistical significance of the univariate tests and multiple regression analyses for electrolytes and renin remained unchanged, although the specific numerical values differed. Consequently, the results presented in this analysis are based on the cohort excluding patients with diabetes. In addition to these exclusions, kidney function and renal diseases were considered, as they are primary factors affecting blood pressure. Kidney function was assessed using the estimated glomerular filtration rate (eGFR). The presence of renal disease was determined based on questionnaire responses. The number of participants with renal disease did not differ significantly between the normal and hypertension groups (*p* = 0.28, Table S9). Similarly, eGFR showed no significant difference between the groups (*p* = 0.10, Figure S1). Consequently, renal disease and eGFR were not included in the multiple regression analysis.

The permutation test revealed statistical differences in effect sizes between sexes. While multiple regression with an interaction term can also detect such differences, it is perhaps better suited as a screening tool, especially when considering the computational demands of permutation. However, for the direct identification of effect sizes within each sex and the significance of their differences, the permutation test is more intuitive and easier to interpret (see Supplementary Results for details).

Sodium and low potassium intakes are known to be associated with hypertension, and sodium is regarded as one of the major causal factors. Previous studies revealed that dietary sodium intake is associated with hypertension [3,6,7]. Clinical trials using sodium dietary restriction showed antihypertensive effects in short-term and long-term follow-up studies [8,9,10]. Low potassium and high sodium consumption or high sodium-to-potassium intake ratios are correlated with a high prevalence of hypertension [8]. In addition, the effects of sodium and potassium can differ between the sexes. For example, potassium intake might have different associations on SBP according to sex, and the hazard ratio of potassium intake on cardiovascular outcomes also differs between males and females [19]. Women showed stronger associations between higher sodium intake and lower sodium-potassium ratio and cardiovascular disease [20]. The 24 h urinary sodium-potassium ratio is correlated with high blood pressure, and this effect is stronger in women [21]. While these results were obtained from the analysis of oral intake or urinary levels of sodium and potassium, even the blood samples used in the measurement of electrolyte levels in our analysis exhibited sex-specific differential associations, especially for sodium levels in women. The blood sodium levels of women showed significant differences between the normal and hypertensive groups in the T-test and multiple logistic regression analysis. However, no significant differences were observed in males. The correlation between blood pressures (SBP and DBP) and sodium levels showed the same tendency. These results strongly indicated that the impact of sodium on the development of hypertension is greater in women. Blood potassium levels showed no significant differences between the total and sex-specific groups regardless of whether the test was performed with single or multiple variables. Although potassium is involved in blood pressure control, serum potassium probably has no independent association with hypertension and blood pressure, considering the current and previous studies [22,23]

Overall, calcium levels showed weak or non-significant results. Significant results were found only in single-variable analyses (Table 1 and Table 2). Although the female group showed a clear significance in the T-test and correlation analysis, the significance tended to be weaker in the multiple regression analysis. In the regression analyses, only nominal significance was observed, and none of the results were significant with multiple testing corrections (Table 3, Table 4 and Table 5). These results were consistent with those of previous studies. Sabanayagam et al. reported that serum calcium levels were significantly associated with hypertension and were more evident in the female population [24], especially aged 40–60 years [25]. Although these results were obtained from different ethnic groups, they are consistent with those of the current study. These results indicated that blood calcium levels might be associated with hypertension in female population.

The results of the chloride level analysis were more complicated. In the single-variable analysis, chloride levels showed a significant correlation only in males (Table 2). Multiple regression analysis revealed that chloride levels had no significant association on hypertension, whereas they had a significant association on DBP in males (Table 5). Although serum chloride levels are predictive of mortality in hypertensive patients [26], chloride levels showed a U-shaped association with the risk of hypertension. However, no explicit analyses of the sex-specific effects of chloride levels on hypertension have been reported. Considering the current results, chloride levels are likely associated with DBP in males only. Additionally, chloride levels may not be associated with hypertension.

Renin showed significant results in all statistical tests using total population and sex-specific groups, which seemed natural because renin is one of the main components involved in the regulation of blood pressure [27]. Notably, renin exhibited suppressive associations (negative effect sizes) in the multiple regression analysis; these were more pronounced in females (Table 3, Table 4 and Table 5), suggesting a potential association with estrogen. Previous research has revealed sex-specific differential effects of renin [28,29,30]. The differential effect of renin is possibly related to estrogen, which prevents angiotensin 2 production [31,32]. Moreover, estrogens, especially estradiol, activate estrogen receptor beta and suppress the synthesis of renin and aldosterone [33,34]. Renin is well-known to induce higher plasma angiotensinogen levels, which results in elevating aldosterone levels that are involved in increasing blood pressure. Taken together, the more pronounced suppressive association between renin and blood pressure may result from estrogen inhibiting the production of angiotensin and aldosterone.

The sex-specific associations observed in women may be primarily explained by the role of estrogen. The findings regarding the relationship between sodium and hypertension are noteworthy because estrogen is known to facilitate sodium excretion through nitric oxide production and subsequent vasodilation [35]. However, it is also well-established that salt sensitivity is greater in the female population [14]. I hypothesize that increased aldosterone production [36] and progesterone-induced upregulation of mineralocorticoid receptor expression [37] may contribute to these findings. Furthermore, chloride may be involved in elevating blood pressure via vascular smooth muscle cell contraction, triggered by chloride-induced membrane depolarization [38]. This mechanism may be linked to the chloride transporter systems TMEM16A (a calcium-activated chloride channel) and NKCC1 (a sodium-potassium-chloride cotransporter) [38]. Previous studies have indicated that these systems are associated with hypertension [39], and estrogen has been shown to modulate these pathways [40,41].

Although age, and BMI were not the main issues in this analysis, sex-specific associations of these factors were identified. BMI was significantly associated with hypertension, but had no significant difference in effect sizes between male and female groups. However, SBP and DBP had significant sex-specific effect sizes. The greater effect size of BMI on DBP in the female group is consistent with a previous report [42]; conversely, the male group being more susceptible to hypertension has also been reported [43]. Moreover, in the adolescent group, male participants showed greater susceptibility to hypertension with changes in BMI [44]. These results were difficult to compare directly because of heterogeneity in the analysis methods and target population. However, considering that the results came from the same East Asian population, sex differences in the effect of BMI on blood pressure may exist.

While sex-specific associations of the hypertension-related factors were identified, this study had some limitations. First, because epidemiologic data from the cross-sectional cohort were included, it was difficult to estimate whether the effects were causal or not. The difficulty in estimating causality is inherent to the cross-sectional design and could be overcome by longitudinal follow-ups. Although the ASAS cohort had longitudinal follow-ups, no information about the variables of this study was available. A longitudinal study might provide more powerful information about causality. Second, serum electrolyte levels are influenced by oral intake and renal excretion. Additionally, certain dietary factors can affect the absorption or excretion of these electrolytes. Unfortunately, because the cohort data lacked detailed information regarding nutritional factors and electrolyte excretion, these variables were not adjusted for in the analysis. Furthermore, serum electrolyte levels are not a direct proxy for salt sensitivity. However, given that significant associations were identified, we can hypothesize that the impact of sodium on systolic blood pressure and hypertension is more pronounced in females. Finally, menopausal status could be an important covariate because estrogen is probably the most critical factor in the estimation of the sex-specific effects. However, as the cohorts had no information about menopausal status, it was not possible to adjust for estrogen depletion effects. These limitations should be considered in further studies to reveal the sex-specific effects on hypertension.

In the results, renin and electrolytes (sodium and chloride) showed differential association between sexes. While statistical significances were obvious, the overall effect sizes were far less than those of the epidemiological factors including age, BMI, and WHR. These should be considered for more effective management strategy of blood pressure. The current results might be applied to the development of sex-specific guidelines for the management of hypertension. For example, a calcium channel blocker with diuretic agents, which are known to be effective for salt-sensitive hypertension, could be prescribed to the female population. Additionally, reducing weight and alcohol consumption could be suggested to male and female patients as a sex-specific management option for hypertension control. Although such guidelines were developed for the general population, they might be applied to specific sex groups selectively [45,46].

## 5. Conclusions

In this study, the sex-specific associations of renin and four electrolytes (sodium, potassium, chloride, and calcium) on hypertension and blood pressure were investigated. For this purpose, a study population with natural physiological status and no therapeutic intervention was used. The results of the analysis using the total population showed findings consistent with those of previous reports. In the analysis of sex-specific subgroups, qualitative and quantitative differences in associations with hypertension or blood pressure were identified using statistical tests. In particular, the renin showed a significant sex-specific association with hypertension. Additionally, well-known factors like sodium and renin also showed differential associations on blood pressure and hypertension, which implies that the sex-specific actions of hypertension-related factors should be evaluated. These results provide invaluable insights into the development of sex-specific management plans for hypertension.

## Figures and Tables

**Table 1:** Comparison of renin and four electrolytes between normal and hypertension groups.

	Population	Normal (*n* = 1237)	HTN (*n* = 6429)	*p* Value
Sodium	Total	142.52 ± 2.16	142.96 ± 2.05	**1.16 × 10** **^−11^**
	Male	142.90 ± 2.08	142.92 ± 2.13	0.80
	Female	142.18 ± 2.18	143.00 ± 1.94	**1.25 × 10^−18^**
Potassium	Total	4.50 ± 0.40	4.52 ± 0.42	1.39 × 10^−1^
	Male	4.56 ± 0.39	4.55 ± 0.42	8.54 × 10^−1^
	Female	4.45 ± 0.41	4.47 ± 0.41	1.68 × 10^−1^
Chloride	Total	103.11 ± 2.30	103.03 ± 2.39	2.23 × 10^−1^
	Male	102.96 ± 2.31	102.74 ± 2.46	3.39 × 10^−2^
	Female	103.25 ± 2.28	103.37 ± 2.25	2.15 × 10^−1^
Calcium	Total	9.58 ± 0.46	9.66 ± 0.49	**3.15 × 10^−7^**
	Male	9.64 ± 0.48	9.70 ± 0.49	**2.81 × 10^−3^**
	Female	9.53 ± 0.44	9.61 ± 0.48	**4.7** **9 × 10^−4^**
Renin	Total	2.67 ± 2.56	2.35 ± 2.74	**1.59 × 10^−^** **^4^**
	Male	3.19 ± 2.77	3.03 ± 3.28	2.61 × 10^−1^
	Female	2.22 ± 2.26	1.51 ± 1.48	**4.49 × 10^−^** **^21^**

*p* values in bold indicate significant results with multiple test corrections (adjusted *p* value = 3.33 × 10^−3^). This is the same with the other tables. HTN, hypertension.

**Table 2:** Comparison of variables between normal and hypertension groups.

Variable	BP	Population	PCC	*p* Value
Sodium	SBP	Total	0.08	**9.51 × 10** **^−1^** **^2^**
		Male	−0.03	4.51 × 10^−2^
		Female	0.15	**5.45 × 10^−^** **^21^**
	DBP	Total	0.11	**3.20 × 10^−22^**
		Male	0.02	1.78 × 10^−1^
		Female	0.14	**9.23 × 10^−^** **^20^**
Potassium	SBP	Total	0.02	8.18 × 10^−2^
		Male	−0.02	2.08 × 10^−1^
		Female	0.03	3.50 × 10^−2^
	DBP	Total	0.04	**1** **.** **2** **2 × 10^−^** **^4^**
		Male	−0.02	2.12 × 10^−1^
		Female	0.06	**3.18 × 10^−4^**
Chloride	SBP	Total	−0.02	1.00 × 10^−1^
		Male	−0.06	**2.84 × 10^−4^**
		Female	0.03	0.1014
	DBP	Total	−0.02	1.10 × 10^−1^
		Male	−0.04	0.01582
		Female	0.03	7.80 × 10^−2^
Calcium	SBP	Total	0.06	**2.59 × 10^−^** **^8^**
		Male	0.04	7.33 × 10^−3^
		Female	0.07	**2.38 × 10^−5^**
	DBP	Total	0.06	**4.19 × 10^−^** **^8^**
		Male	−0.04	1.09 × 10^−2^
		Female	0.03	7.37 × 10^−2^
Renin	SBP	Total	−0.09	**1.99 × 10^−^** **^16^**
		Male	−0.07	**3.02 × 10^−^** **^5^**
		Female	−0.17	**3.80 × 10^−^** **^2^** **^6^**
	DBP	Total	−0.04	**1.48 × 10^−^** **^3^**
		Male	−0.03	5.00 × 10^−2^
		Female	−0.12	**1.61 × 10^−15^**

BP, blood pressure; PCC, Pearson correlation coefficient; SBP, systolic blood pressure; DBP, diastolic blood pressure.

**Table 3:** Sex-specific multiple logistic regression analysis with covariates.

	Male			Female		
Variables	Estimate (SE)	z Value	*p* Value	Estimate (SE)	z Value	*p* Value
Age	0.04 (0.01)	7.33	**2.32 × 10^−13^**	0.06 (0.01)	9.39	**5.82 × 10^−21^**
BMI	0.06 (0.02)	3.35	**8.04 × 10^−4^**	0.09 (0.02)	5.70	**1.23 × 10^−8^**
WHR	5.64 (0.92)	6.14	**8.10 × 10^−10^**	3.06 (0.69)	4.42	**9.83 × 10^−6^**
Albumin	0.51 (0.19)	2.66	7.72 × 10^−3^	0.40 (0.24)	1.67	9.55 × 10^−2^
BUN	−0.01 (0.01)	−1.04	2.99 × 10^−1^	−0.02 (0.02)	−1.55	1.22 × 10^−1^
Creatinine	−0.50 (0.33)	−1.52	1.28 × 10^−1^	−0.23 (0.48)	−0.48	6.30 × 10^−1^
Protein	0.27 (0.14)	1.93	5.37 × 10^−2^	0.47 (0.16)	2.93	3.34 × 10^−3^
Ca	0.21 (0.10)	2.03	4.27 × 10^−2^	0.20 (0.12)	1.70	8.95 × 10^−2^
Na	0.05 (0.03)	1.80	7.23 × 10^−2^	0.14 (0.03)	4.88	**1.04 × 10^−6^**
K	−0.06 (0.11)	−0.51	6.09 × 10^−1^	−0.17 (0.12)	−1.43	1.53 × 10^−1^
CL	−0.04 (0.02)	−1.48	1.39 × 10^−1^	−0.06 (0.03)	−2.27	2.30 × 10^−2^
Renin	−0.03 (0.02)	−2.05	4.02 × 10^−2^	−0.21 (0.03)	−5.99	**2.06 × 10^−9^**
Smoke	−0.21 (0.11)	−1.91	5.63 × 10^−2^	0.02 (0.23)	0.10	9.20 × 10^−1^
Drink	0.49 (0.12)	3.92	**8.81 × 10^−5^**	0.03 (0.11)	0.23	8.20 × 10^−1^
fhHTN	0.33 (0.12)	2.79	5.24 × 10^−3^	0.28 (0.13)	2.14	3.26 × 10^−2^

SE, standard error; z value, z statistics from multiple linear regression; WHR, waist-to-hip ratio; fhHTN, family history of hypertension; BMI, body mass index; BUN, blood urea nitrogen; Ca, calcium; Na, sodium; K, potassium; CL, chloride. The notation for the electrolytes is the same in the other tables.

**Table 4:** Multiple linear regression analysis of SBP and covariates.

	Male			Female		
Variables	Estimate (SE)	t Value	*p* Value	Estimate	t Value	*p* Value
Age	0.51 (0.03)	15.92	**2.87 × 10^−55^**	0.67 (0.03)	19.81	**2.09 × 10^−83^**
BMI	0.37 (0.10)	3.61	**3.07 × 10^−4^**	0.69 (0.08)	8.39	**6.82 × 10^−17^**
WHR	46.48 (5.03)	9.24	**3.89 × 10^−20^**	28.65 (3.44)	8.32	**1.21 × 10^−16^**
Albumin	1.45 (1.04)	1.39	1.64 × 10^−1^	1.20 (1.16)	1.03	3.02 × 10^−1^
BUN	−0.03 (0.07)	−0.43	6.66 × 10^−1^	−0.21 (0.07)	−2.76	5.80 × 10^−3^
Creatinine	−5.09 (1.77)	−2.88	3.94 × 10^−3^	−1.69 (1.99)	−0.85	3.95 × 10^−1^
Protein	2.54 (0.75)	3.38	**7.44 × 10^−4^**	3.14 (0.75)	4.20	**2.68 × 10^−5^**
Ca	1.03 (0.55)	1.87	6.15 × 10^−2^	1.14 (0.58)	1.96	4.98 × 10^−2^
Na	0.28 (0.14)	2.01	4.43 × 10^−2^	0.44 (0.13)	3.35	**8.08 × 10^−4^**
K	−1.22 (0.62)	−1.98	4.77 × 10^−2^	−1.15 (0.60)	−1.94	5.30 × 10^−2^
CL	−0.34 (0.13)	−2.63	8.61 × 10^−3^	−0.30 (0.12)	−2.39	1.68 × 10^−2^
Renin	−0.44 (0.09)	−5.19	**2.25 × 10^−7^**	−0.94 (0.11)	−8.46	**3.57 × 10^−17^**
Smoke	−1.33 (0.63)	−2.12	3.39 × 10^−2^	−1.12 (1.10)	−1.02	3.07 × 10^−1^
Alcohol	2.45 (0.62)	3.98	**6.94 × 10^−5^**	−0.73 (0.53)	−1.37	1.70 × 10^−1^
fhHTN	2.13 (0.66)	3.20	**1.39 × 10^−3^**	2.64 (0.63)	4.21	**2.64 × 10^−5^**

SE, standard error; WHR, waist-hip ratio; fhHTN, family history of hypertension; BMI, body mass index; Drink, alcohol comsumption; BUN, blood urea nitrogen.

**Table 5:** Result from multiple linear regression analysis of DBP and covariates.

	Male			Female		
Variables	Estimate	t Value	*p* Value	Estimate	t Value	*p* Value
Age	0.16 (0.02)	7.20	**7.36 × 10^−13^**	0.25 (0.02)	11.21	**9.53 × 10^−29^**
BMI	0.44 (0.07)	6.24	**4.96 × 10^−10^**	0.55 (0.05)	10.18	**4.80 × 10^−24^**
WHR	32.21 (3.43)	9.38	**1.08 × 10^−20^**	23.22 (2.25)	10.32	**1.23 × 10^−24^**
Albumin	2.52 (0.71)	3.53	**4.16 × 10^−4^**	3.10 (0.76)	4.08	**4.62 × 10^−5^**
BUN	−0.08 (0.05)	−1.70	8.98 × 10^−2^	−0.14 (0.05)	−2.77	5.68 × 10^−3^
Creatinine	−1.69 (1.21)	−1.40	1.61 × 10^−1^	0.59 (1.30)	0.46	6.48 × 10^−1^
Protein	1.42 (0.51)	2.76	5.76 × 10^−3^	1.40 (0.49)	2.87	4.13 × 10^−3^
Ca	0.06 (0.37)	0.16	8.72 × 10^−1^	0.46 (0.38)	1.21	2.28 × 10^−1^
Na	0.32 (0.09)	3.42	**6.39 × 10^−4^**	0.41 (0.09)	4.82	**1.46 × 10^−6^**
K	−0.44 (0.42)	−1.05	2.93 × 10^−1^	0.18 (0.39)	0.47	6.40 × 10^−1^
CL	−0.29 (0.09)	−3.36	**7.78 × 10^−4^**	−0.17 (0.08)	−2.11	3.48 × 10^−2^
Renin	−0.19 (0.06)	−3.34	**8.58 × 10^−4^**	−0.47 (0.07)	−6.43	**1.46 × 10^−10^**
Smoke	−0.90 (0.43)	−2.10	3.56 × 10^−2^	−0.84 (0.72)	−1.16	2.45 × 10^−1^
Drink	2.07 (0.42)	4.92	**8.86 × 10^−7^**	0.22 (0.35)	0.65	5.19 × 10^−1^
fhHTN	1.39 (0.45)	3.07	**2.14 × 10^−3^**	1.23 (0.41)	2.99	**2.80 × 10^−3^**

WHR, waist-hip ratio; Alcohol, alcohol consumption; SE, standard error; BMI, body mass index; fhHTN, family history of hypertension; BUN, blood urea nitrogen.

## Data Availability

The original data presented in the study are openly available in CODA (https://coda.nih.go.kr/, accessed on 26 December 2023).

## Supplementary Materials

The following supporting information can be downloaded at: https://www.mdpi.com/article/10.3390/jcm15020643/s1, Table S1: proportion of kidney disease in normal and hypertension group; Table S2: multiple logistic regression analysis of hypertension in total population; Table S3: linear regression analysis of SBP in total population; Table S4: linear regression analysis of DBP in total population; Table S5: result of interaction analysis, Table S6: result of multiple logistic regression for hypertension incorporating interaction of sex, Table S7: result of multiple linear regression for SBP incorporating interaction of sex, Table S8: result of multiple linear regression for DBP incorporating interaction of sex, Table S9: 2-by-2 table of hypertension and renal diseases; Figure S1: comparison of eGFR between normal and hypertension group [47].

## Abbreviations

The following abbreviations are used in this manuscript:

KoGES	Korean Genome and Environmental Study
BMI	Body mass index
WHR	Waist-to-hip ratio
SBP	Systolic blood pressure
DBP	Diastolic blood pressure
ASAS	Ansan-Ansung
CC	Correlation coefficient
