# Supplementary material for: A Cross-Sectional Study of Sex-Specific Associations of Renin and Electrolytes on the Development of Hypertension
Source: J Clin Med. 2026 Jan 13;15(2):643. doi: 10.3390/jcm15020643 (PMC12842152; doi:10.3390/jcm15020643)
Supplement: Supplementary file 1 [file jcm-15-00643-s001.zip › Supplementary Methods and Results.pdf]

Supplementary Methods

Measurement of renin and electrolytes

After at least 8 hours of fasting, all blood samples were obtained in the morning. Renin was sampled with the participant in an upright position and measured using an immunoradiometric assay (device: Gamma Counter Cobra, Packard, USA; reagent: INS-IRMA, SRL, Japan). Three electrolytes—sodium, potassium, and chloride—were measured by the Integra 800 (Roche, USA) and ISE Buffer (Roche, USA). Calcium was measured by colorimetry (device: HITACHI Auto Analyzer 7600 (Hitachi, Japan); reagent: Calcium (Ca) (EIKEN, Japan)).

Multiple regression analysis with interaction

The regression analysis is determining the regression equation with set of independent predictor variables (xi) and dependent variables (y) as follows.

$$Y \sim a1*x1+a2*x2+a3*x3.....$$

In the model described above, ai denotes the regression coefficient, representing the marginal effect of the variable on the dependent variable. By adding an interaction term (ak*xi:xj, a4 is coefficient), we can determine if the relationship between a predictor and the outcome depends on the status of another predictor. In linear regression, this indicates that the slope of one predictor varies according to the value of the other. Similarly, in a logistic regression model, an interaction implies that the logistic curve for one predictor changes its shape depending on the level of the other predictor.

For interaction analysis of sex effect on hypertension, the model was determined as follows.

HTN ~ age + sex + BMI + WHR + albumin + protein + BUN + Creatinine + calcium + sodium + potassium + chloride + renin + sex:target_variable

In the above equation, the target variables were those that showed significant results in the permutation analysis or exhibited discordant significance between the sexes. In addition to hypertension, SBP and DBP were also used as dependent variables in the model.

Multiple testing correction with Bonferroni's method

The Bonferroni correction was used to control the family-wise error rate (FWER) during multiple testing. It is well known that multiple hypothesis testing increases the false positive rate. This probability, the FWER, is estimated as $1-(1-\alpha)^k$, where α is the significance level and k is the number of tests. The FWER increases as k grows. To mitigate this, the Bonferroni method adjusts the significance threshold to α/k. By lowering the P value threshold in this manner, the FWER can be effectively controlled.

Supplementary Results

Result of Interaction analysis

In the interaction analysis, variables exhibiting discordant significance between sex-stratified groups often showed high significance. For example, in the multiple logistic regression of hypertension, the effect sizes of sodium and renin were significant in the female group only (Table 3), and the interaction analysis yielded significant results after multiple testing correction (Table S5). While sodium showed no significant result in the interaction analysis (Table 4), alcohol consumption was significantly associated with SBP in the multiple linear regression (Table S5). In the multiple linear regression of DBP, alcohol consumption and chloride exhibited sex-specific significance (Table 5); however, only alcohol consumption showed a significant result in the interaction analysis. It appears that even when results are discordant in sex-specific analyses, the interaction analysis may not reach significance if the differences in effect sizes are not substantial. Additionally, some variables exhibited both marginal and interaction effects in the logistic regression models (Tables S6, S7, and S8). These findings could be applied to identify variables possessing both marginal and interaction effects, which may have novel biological implications for the development of hypertension.

Table S5. Result of interaction analysis

Dependent variable	variable	Estimate	Standard error	Statistics	P value
**Hypertension**	sex:age	0.02	0.01	2.52	1.16E-02
	sex:BMI	0.00	0.02	0.06	9.56E-01
	sex:WHR	-1.52	0.97	-1.56	1.19E-01
	sex:Na	0.11	0.03	3.21	**1.33E-03**
	sex:renin	-0.18	0.04	-4.85	**1.25E-06**
	sex:drink	-0.52	0.16	-3.16	**1.58E-03**
**Systolic blood pressure**	age:sex	0.12	0.04	2.85	**4.42E-03**
	sex:BMI	0.13	0.11	1.11	2.66E-01
	sex:WHR	-6.79	5.03	-1.35	1.77E-01
	sex:protein	0.39	0.77	0.50	6.16E-01
	sex:Na	0.34	0.16	2.13	3.34E-02
	sex:renin	-0.55	0.14	-4.03	**5.74E-05**
	sex:drink	-3.55	0.80	-4.44	**9.24E-06**
	sex:fhHTN	0.38	0.91	0.42	6.76E-01
**Diastolic blood pressure**	sex:age	0.08	0.03	2.84	**4.54E-03**
	sex:BMI	0.01	0.08	0.09	9.28E-01
	sex:WHR	-4.06	3.35	-1.21	2.26E-01
	sex:albumin	0.38	0.75	0.50	6.16E-01

	sex:Na	0.27	0.11	2.47	1.34E-02
	sex:CL	0.20	0.10	2.03	4.21E-02
	sex:renin	-0.29	0.09	-3.24	**1.20E-03**
	sex:drink	-2.07	0.53	-3.88	**1.03E-04**
	sex:fhHTN	-0.29	0.61	-0.48	6.34E-01

Na: sodium, CL: chloride, fhHTN: family history of hypertension, BMI: body mass index, WHR: waist hip ratio.

Interaction analysis within the multiple regression model can reveal differences in effect sizes across groups. Therefore, in addition to the permutation test, an interaction analysis was performed to identify the differential effects of the variables on blood pressure and hypertension. The results of the interaction analysis were generally consistent with those of the permutation test, although age and BMI yielded different results (Table S5). In cases where statistical significance was observed in only one sex group, the interaction analysis typically showed high significance. For example, variables identified as sex-specific in the multiple logistic regression analysis (e.g., sodium, renin, and alcohol consumption) also yielded significant results in the interaction analysis (Table S5). While interaction analysis identifies significant differences in the associations between variables and outcomes, it does not provide information regarding the statistical significance of those variables within each individual sex group. Therefore, although the interaction analysis identified sex-specific effects, the permutation test was necessary to accurately estimate the group-specific effects and their associated significance levels. However, interaction analysis remains a valuable screening tool for identifying the differential effects of certain factors. Additionally, some variables such as age, alcohol consumption, and sodium exhibited both marginal and interaction effects in the logistic regression models (Tables S6, S7, and S8). These findings could be applied to identify variables possessing both marginal and interaction effects, which may have novel biological implications for the development of hypertension.

Comparison of kidney function

In the comparison of variables between the normal and hypertension groups, kidney disease status and estimated glomerular filtration rate (eGFR) were compared to identify possible bias due to kidney conditions. The difference in kidney disease was not significant between the normal and hypertension groups (Chi-square test P = 0.28, Table S9). Moreover, the comparison of eGFR showed no significant difference (t-test P = 0.10, Figure S1). Given these results, it seemed that no significant change in kidney function was identified between the groups. The eGFR was determined by the Cockcroft-Gault equation.

Table S9. **2-by-2 table of hypertension and kidney disease.** Each cell contains number of participants that are matched to the statuses of hypertension and kidney disease.

	Kidney disease (-)	Kidney disease (+)
Hypertension (-)	6451	143
Hypertension (+)	1203	34

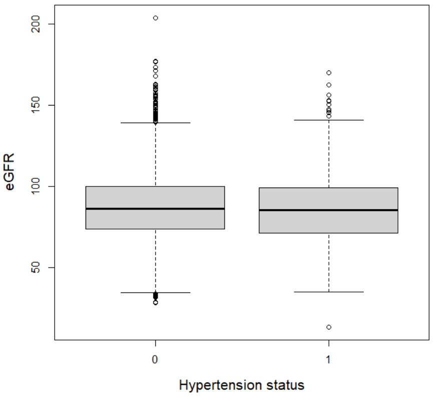

Figure S1. Comparison of eGFR between normal and hypertension group. eGFR: estimated glomerular filtration rate

Reference

Ranstam J. Multiple P-values and Bonferroni correction. Osteoarthritis Cartilage. 2016 May;24(5):763-4. doi: 10.1016/j.joca.2016.01.008. Epub 2016 Jan 21. PMID: 26802548.
